# Human Rickettsiosis Caused by *Rickettsia parkeri* Strain Atlantic Rainforest, Urabá, Colombia

**DOI:** 10.3201/eid2612.200388

**Published:** 2020-12

**Authors:** Margarita Arboleda, Leidy Y. Acevedo-Gutiérrez, Alejandra Ávila, Dairo Ospina, Francisco J. Díaz, David H. Walker, Juan D. Rodas

**Affiliations:** Instituto Colombiano de Medicina Tropical–CES, Medellín, Colombia (M. Arboleda);; Universidad de Antioquia, Medellín (L.Y. Acevedo-Gutiérrez, F.J. Díaz, J.D. Rodas);; Clínica Aurora y Universidad Pontificia Bolivariana, Medellín (A. Ávila); Clínica Chinita, Apartadó, Colombia (D. Ospina);; University of Texas Medical Branch, Galveston, Texas, USA (D. Walker)

**Keywords:** tickborne diseases, febrile syndrome, emerging diseases, tropical medicine, rickettsia, Rickettsia parkeri, bacteria, Colombia

## Abstract

We describe the clinical, serologic, and molecular findings of a new human rickettsiosis in Colombia. Antibodies against *Rickettsia* spp. were detected. PCR showed amplification of genes for *R. parkeri* strain Atlantic Rainforest. This new rickettsiosis of minor virulence could explain some of the undifferentiated acute febrile diseases in Colombia.

Among the numerous causes of acute undifferentiated nonmalarial febrile illness, rickettsiae are amenable to treatment that can prevent death or, in the case of non–life-threatening diseases, shorten and ameliorate the course of illness (*1*). Awareness and knowledge of these infectious diseases are crucial and necessary. In Colombia, Rocky Mountain spotted fever was recognized in the 1930s and then rediscovered in the 21st century (*2*). Clusters of cases were documented in the departments of Cundinamarca, Córdoba, and Antioquia (*2*–*4*). Five fatal cases of Rocky Mountain spotted fever occurred in the village of Las Changas in the district of Necoclí in 2006, and 4 fatal cases occurred in a village in the district of Turbo in 2008 (*4*). Prevalence of antibodies to spotted fever group (SFG) rickettsiae of 25.6% among healthy residents of several areas in Colombia suggests contact of persons with less-virulent SFG rickettsiae, such as *Rickettsia parkeri*, which has previously been reported in Colombia in ticks of the species *Amblyomma ovale*, and *R. amblyommatis,* previously reported in *A. cajennense* ticks (*5*–*7*).

## The Case

A previously healthy 47-year-old male farmer who lived in a rural area near the village of Puerto López in the district of Turbo, Department of Antioquia, Colombia, came to a clinic in Apartadó with a 5-day history of fever. He also reported chills, asthenia, nausea, loss of appetite, dark urine, and dysuria that began 1 day after discovery of a tick on the left flank of his abdomen. On physical examination he appeared to be in generally good condition; no rash was identified, and he was afebrile with normal vital signs.

A 13 × 16 mm crust with an erythematous halo and scaling collaret was seen above the left iliac crest at the site of the tick bite, accompanied by ipsilateral visible tender inguinal lymphadenopathy 3.5 cm in length (Figure). Results of the patient’s complete blood counts were within reference ranges, and blood smear detected no plasmodia.

**Figure:**
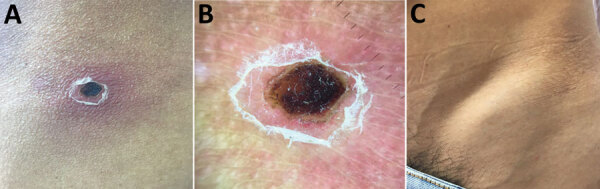
Eschar of inoculation and lymphadenopathy in patient with febrile syndrome caused by a strain of *Rickettsia parkeri*, Colombia*.* A) A 1 .3 × 1.6 cm eschar with an erythematous halo is present at the site of a tick bite above the left iliac crest. B) A closer view reveals a brown-black crust and a collarette of desquamation. C) Distended skin overlying enlarged draining lymph nodes, 3.5 cm in length, is visible in the left inguinal region.

Because of the eschar, draining lymphadenopathy, and history of tick bite, a rickettsial infection was suspected, and the patient was referred to the Colombian Institute of Tropical Medicine in Apartadó, Antioquia. There, a punch biopsy of the inoculation eschar and scraping of the eschar base and blood samples were obtained, and treatment with doxycycline (200 mg/d for 7 d) was initiated. The patient subsequently reported defervescence after 48 hours, with disappearance of symptoms and decreased lymphadenopathy.

The patient’s serum samples were examined by indirect immunofluorescence assay for antibodies reactive with 6 rickettsiae: *R. rickettsii* strain Uramita, *R. parkeri* strain At24, *R. amblyommatis* strain Ac37, *R. rhipicephali* strain HJ5, *R. bellii* strain Mogi, and *Candidatus* Rickettsia colombianensi (slides donated by Marcelo Labruna, Faculdade de Medicina Veterinária e Zootecnia, Universidade de São Paulo, São Paulo, Brazil). DNA was extracted from the eschar crust, eschar biopsy, and scrapings with the DNeasy Blood and Tissue kit (QIAGEN, https://www.qiagen.com), according to the manufacturer’s instructions. The samples were processed by PCR for *gltA*, *sca0*, *sca4*, and *sca5* genes of *Rickettsia* using previously described primers and methods (*8*). The amplification products were sent for sequencing to Macrogen, and the sequences were analyzed with BLASTn (https://blast.ncbi.nlm.nih.gov), MEGA 7.0 (https://www.megasoftware.net*)*, and MrBayes 3.2.6 (https://www.geneious.com). The human protocol was approved by the Bioethical Committee at the University of Antioquia (act 001/2016).

Indirect fluorescent antibody testing demonstrated seroconversion to all 6 *Rickettsia* antigens. Acute serum samples showed reactivity with none of the antigens at a titer of 64, whereas seroconversion to each of the antigens was observed by reactivity with *R. parkeri* (titer 1,024); *R. rickettsii* (titer 512); *R. bellii* (titer 256); and *R. amblyommatis*, *Candidatus* R. colombianensi, and *R. rhipicephali* (titer 128), without a quadruple difference between the different antigens.

Amplicons were obtained for all genes tested and all samples analyzed. All the sequences demonstrated 100% identity with *R. parkeri* strain Atlantic Rainforest and were submitted to GenBank (accession nos. MK860199–202).

Previous patients with rickettsial infections in the Department of Antioquia were gravely ill with rapid deterioration and respiratory and renal compromise, hepatic injury, and case-fatality rates of 28%–35% (*4*). In contrast, *R. parkeri* infections are mild or moderate acute febrile illnesses with fever, chills, headache, arthromyalgia, inoculation eschar, maculopapular or vesicular rash, and painful lymphadenopathy at lymphatic drainage sites of the entry lesion. Some patients do not manifest lymphadenopathy or, as in this patient, a rash (*9*).

In 2004, a new SFG human infection attributed to *R. parkeri* sensu stricto was described in the United States (*10*); later, Spolidorio et al. described a patient in Brazil with a clinically identical moderate febrile condition, with myalgia, arthralgia, and eschar, caused by a strain of *R. parkeri* designated Atlantic Rainforest (*11*). Human infections with similar signs and symptoms caused by *R. parkeri* sensu stricto and *R. parkeri* strain Atlantic Rainforest have been documented in the United States, Brazil, Uruguay, Argentina, and now in Colombia; meanwhile, these bacteria have also been demonstrated in ticks (in the absence of recognized human infections) in Bolivia, Peru, Nicaragua, Belize, and Mexico. *Amblyomma maculatum*, *A. triste*, and *A. tigrinum* ticks have been found infected with *R. parkeri* sensu stricto in nature; *R. parkeri* strain Atlantic Rainforest has been reported in *A. ovale, A. aureolatum*, and *Dermacentor parumapertus* ticks (*12*,*13*). *R. parkeri* strain Atlantic Rainforest was previously identified in *A. ovale* ticks in Colombia, where dogs are suspected of bringing the ticks into homes (*6*).

## Conclusions

There may be a substantial unrecognized occurrence of *R. parkeri* infections in South and Central America. Increased awareness and knowledge by primary care physicians and establishment of effective national surveillance programs and guidelines, including empiric treatment with doxycycline, would lead to improved patient outcomes. Enhanced research to identify the range of vectors, vertebrate hosts, and risk factors to predict human exposure to this and other agents that may be causing similar diseases would contribute to the elucidation of the causes of acute febrile illnesses in Latin America.
